# Ras Downstream Effector GGCT Alleviates Oncogenic Stress

**DOI:** 10.1016/j.isci.2019.07.036

**Published:** 2019-07-26

**Authors:** Zaoke He, Shixiang Wang, Yuanyuan Shao, Jing Zhang, Xiaolin Wu, Yuxing Chen, Junhao Hu, Feng Zhang, Xue-Song Liu

**Affiliations:** 1 School of Life Science and Technology, ShanghaiTech University, Shanghai 201203, China; 2 Core Facility, Department of Clinical Laboratory, Quzhou People's Hospital, Quzhou, Zhejiang, China; 3 Interdisciplinary Research Center on Biology and Chemistry, Shanghai Institute of Organic Chemistry, Chinese Academy of Sciences, Shanghai, China; 4 Shanghai Institute of Biochemistry and Cell Biology, Chinese Academy of Sciences, Shanghai, China; 5 University of Chinese Academy of Sciences, Beijing, China

**Keywords:** Biological Sciences, Cell Biology, Cancer

## Abstract

How cells adapt to oncogenic transformation-associated cellular stress and become fully transformed is still unknown. Here we identified a novel GGCT-regulated glutathione (GSH)-reactive oxygen species (ROS) metabolic pathway in oncogenic stress alleviation. We identified GGCT as a target of oncogenic Ras and that it is required for oncogenic Ras-induced primary mouse cell proliferation and transformation and *in vivo* lung cancer formation in the *LSL-Kras G12D* mouse model. However, *GGCT* deficiency is compatible with normal mouse development, suggesting that GGCT can be a cancer-specific therapeutic target. Genetically amplified *GGCT* locus further supports the oncogenic driving function of GGCT. In summary, our study not only identifies an oncogenic function of GGCT but also identifies a novel regulator of GSH metabolism, with implications for further understanding of oncogenic stress and cancer treatment.

## Highlights

• GGCT is a target of Ras and is required for Ras-induced cancer formation
• *GGCT* deletion is compatible with normal mouse development and tissue function
• *GGCT* genomic locus is amplified in multiple human cancer types
• GGCT could alleviate oncogenic stress by regulating GSH-ROS metabolism

## Graphical Abstract

**Figure:**
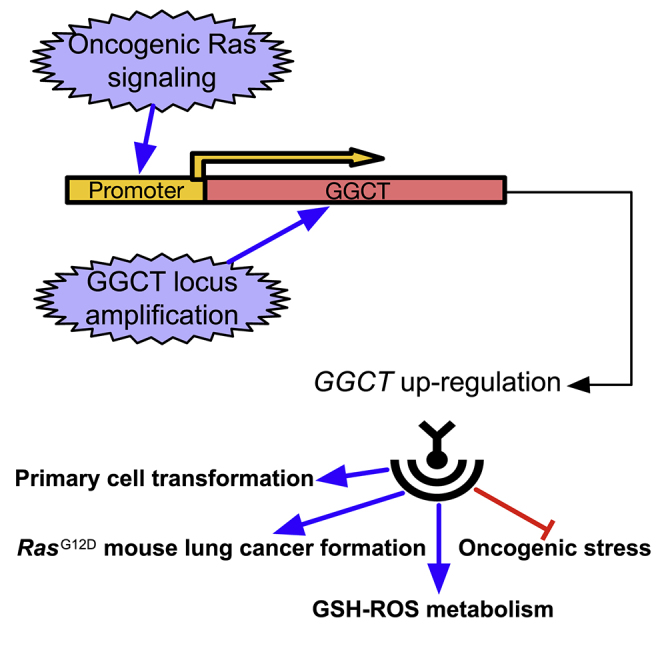

## Introduction

Oncogenic transformation in primary somatic cells always leads to cellular stresses, which function as a fail-safe mechanism to prevent cancer formation (Luo et al., 2009). How cells adapt to these oncogenic stresses and become fully transformed is still not very clear. Activating Ras mutations are frequently observed in various cancers; however, these Ras mutants are known to be “undruggable” targets (Cox et al., 2014). Ras downstream targets would be surrogate drug targets for these Ras oncoproteins. Here we identified a novel oncogenic Ras downstream target GGCT, and further characterized GGCT function using mouse models, cancer genomics, and cell biochemical approaches.

GGCT was previously named C7orf24 and was originally identified as a protein up-regulated in bladder urothelial carcinoma (Kageyama et al., 2007). Subsequent studies indicated that GGCT protein or mRNA is overexpressed in multiple human cancers including breast (Gromov et al., 2010), lung, esophagus, stomach, bile duct, and uterine cervix cancer (Amano et al., 2012). In 2008, C7orf24 was identified as γ-glutamyl cyclotransferase, and this study renamed C7orf24 as GGCT (Oakley et al., 2008). The physiological function of this enzyme activity in mammals is not clear. Actually, C7orf24 is not the only protein showing this enzyme activity in mammalian cells (Chi et al., 2014). γ-Glutamyl cyclotransferase catalyzes the following reaction: γ-glutamyl-amino acid → 5-oxoproline + amino acid. This enzyme was supposed to participate in glutathione (GSH) homeostasis. Extracellular GSH can be hydrolyzed by membrane-bound γ-glutamyl transpeptidase (GGT) to cysteinylglycine and γ-glutamyl-amino acid dipeptide (Anderson, 1998, Meister, 1988). In the cytoplasm, γ-glutamyl cyclotransferase cleaves the γ-glutamyl-amino acid to give 5-oxoproline and amino acid (Meister, 1974). However, the function of GGCT (C7orf24) in GSH homeostasis is still unknown. The function of γ-glutamyl cyclotransferase enzyme activity in cancer is also unknown; association between this enzyme activity and human cancer has not been reported.

It is already known that protein and mRNA expression of *GGCT* is up-regulated in multiple types of cancers. However, it is still unknown if *GGCT* expression up-regulation is simply a by-product of cancer formation or if *GGCT* up-regulation is required for cancer evolution. The selective accumulation of genetic alterations favoring *GGCT* up-regulation in cancer, but not normal control tissues, can serve as important cancer genomic evidence supporting the cancer-driving (or oncogenic) function of *GGCT*.

Here we systematically studied human cancer genome and identified significant *GGCT* gene amplification in human lung adenocarcinoma (LUAD). *GGCT* genomic locus amplification can directly lead to *GGCT* mRNA up-regulation, suggesting a cancer-driving function of *GGCT* in human cancer. With newly generated *GGCT* knockout mouse model and primary cells, we demonstrated a critical role of GGCT in GSH homeostasis and redox balance, critical for primary cell transformation and lung cancer formation, but not normal mouse development.

## Results

### Chromosome 7p Amplification and Associated Prognosis in Human Lung Adenocarcinoma (LUAD)

Genetic alterations including point mutations and copy number variations in somatic cells are the driving forces for human cancer. Recent cancer genomics efforts, such as The Cancer Genome Atlas (TCGA), enable us to systematically study the genetic alterations in cancer, and many novel oncogenes or tumor suppressors have been identified in this way (Kandoth et al., 2013, Zack et al., 2013). Here we focused on the copy number alterations of human LUAD, and observed that the short arm of chromosome 7 (7p) is among the top amplified chromosome fragments based on several independent studies (Balsara and Testa, 2002, Lu et al., 2011, Ni et al., 2013, Weir et al., 2007, Wu et al., 2015) (Figure S1A). Specifically, 53% of LUAD has amplified 7p (Table S1). In total 389 genes are located in human chromosome 7p region. In addition to LUAD, chromosome 7p is also amplified in other types of human cancers, including colon cancer, glioblastoma, prostate cancer, etc. (Table S1).

To investigate whether 7p amplification has an impact on the prognosis of patients with LUAD, we compared the survival curves of patients with LUAD with and without chromosome 7p amplification and observed that patients with LUAD with chromosome 7p amplification were significantly associated with poor prognosis compared with patients without 7p amplification (Figure S1B). In early-stage (TNM stage I) LUAD, the effect of 7p amplification on the prognosis is statistically significant, whereas in the late stages (TNM stage III and IV), 7p amplification is not significantly associated with poor patient prognosis (Figure S1C). This implicates a specific function of 7p amplification in early-stage LUAD.

As 7p is widely amplified in human cancer, some oncogenes located in 7p may be co-amplified and their expression up-regulated consequently. To identify these potential oncogenes in 7p region, we systematically compared the mRNA expression of 389 chromosome 7p genes in normal lung and LUAD samples (Table S2). *GGCT* was among the top significantly up-regulated genes when we compare LUAD with normal lung samples. This implies that *GGCT* may be one of the target genes responsible for 7p amplification-associated cancer. *GGCT* chromosome locus 7p14.3 was reported to be amplified in lung cancer (Choi et al., 2007), and in the 7p14.3 region, GGCT is the top significantly expressed up-regulated gene, suggesting that *GGCT* could also be the target of 7p14.3 amplification in lung cancer.

### Stimulation of *GGCT* Transcription by Activated Ras Signaling

**Figure 1:**
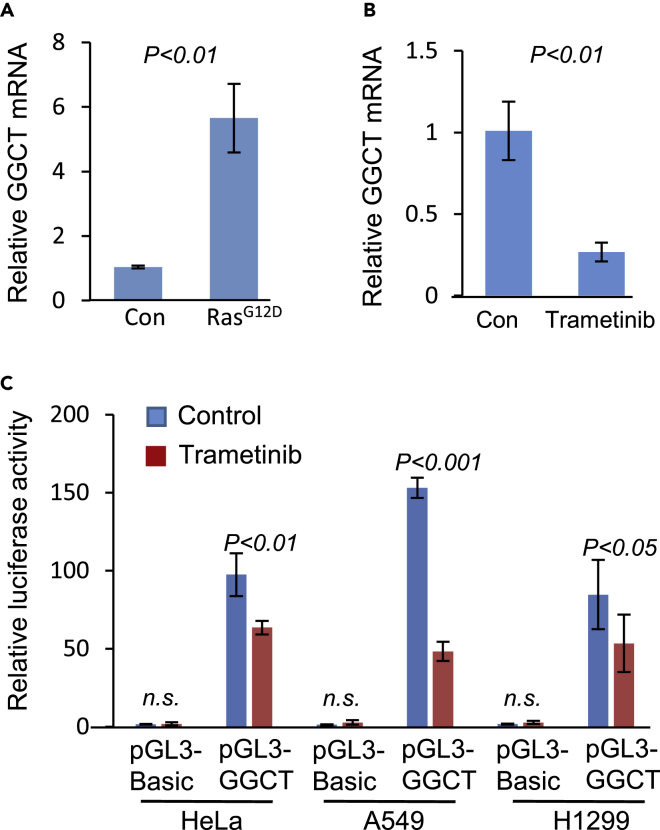
RAS Regulates *GGCT* Transcription (A) Constitutively active *RAS* (*KRAS*^*G12D*^) expression stimulates *GGCT* transcription in primary mouse embryonic fibroblasts (MEFs). Error bars represent mean ± SD from three experiments. (B) Trametinib (MEK inhibitor) inhibits *GGCT* transcription in MEFs. *GGCT* mRNA was detected by real-time PCR. Error bars represent mean ± SD from three experiments. (C) Luciferase reporter assay was performed in HeLa, A549, and H1299 cells with human *GGCT* promoter driving pGL3 vector in the presence or absence of MEK inhibitor trametinib. Significantly decreased *GGCT* promoter, but not pGL3-basic promoter, activity was observed in the presence of trametinib. Error bars represent mean ± SD of three experiments. p Values of unpaired two-tailed t test are shown.

*GGCT* gene was originally identified when we compared the differentially expressed genes between *Kras*^*G12D*^-expressing and control primary mouse embryonic fibroblasts (MEFs) (Figure 1A). When Ras signaling was inhibited with MEK inhibitor trametinib (0.5 μM, 24 h), we observed that *GGCT* mRNA expression was down-regulated (Figure 1B). These observations implicate that *GGCT* mRNA expression is under the regulation of oncogenic growth signaling. We cloned human *GGCT* promoter and used it to drive the transcription of luciferase reporter gene. In the presence of trametinib (0.5 μM, 24 h), *GGCT* promoter activity is also significantly decreased (Figure 1C). To further validate the induction of GGCT by Ras signaling, we knocked down *KRAS* gene in human cancer cells and observed that *GGCT* transcription is down-regulated (Figure S2). These studies imply that oncogenic Ras signal transcriptionally regulates *GGCT* expression. When combined with the observation that *GGCT* locus is amplified in cancer, *GGCT* transcription regulation by Ras oncogenic signal further supports cancer-related function of *GGCT*.

### *GGCT* CNV Amplification in Human Cancer

**Figure 2:**
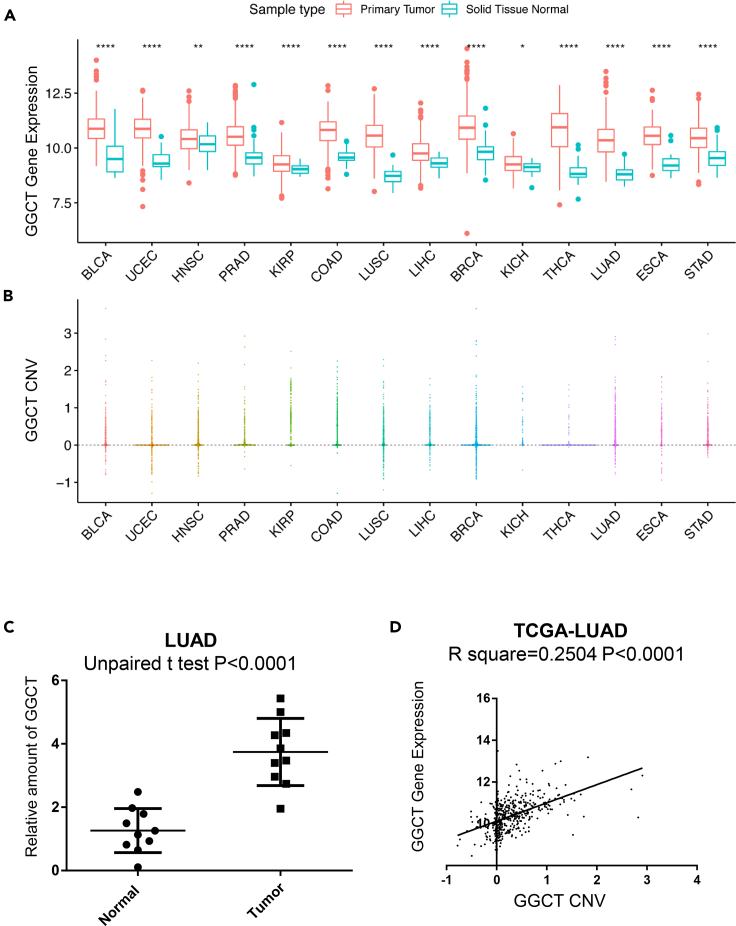
*GGCT* Copy Number Variation (CNV) and mRNA Expression Status in Human Cancers (A) *GGCT* mRNA expression levels (log2 based) were statistically up-regulated (unpaired two-tailed t test) in 14 of 15 types of human cancers when compared with corresponding normal control tissues based on The Cancer Genome Atlas (TCGA) database. Only 15 of 32 TCGA cancer types have both tumor and normal control samples available, and the number of normal samples is greater than or equal to 10. ∗∗∗∗p < 0.0001, ∗∗p < 0.01, ∗p < 0.05. (B) *GGCT* copy number values (log2 based ratio, normal copy number is 0) obtained from GISTIC2 software in cancers as (A) are shown based on TCGA database. GISTIC2 CNV value 0 means normal copy number. (C) *GGCT* CNV values were detected by qPCR in patients with LUAD (n = 10) and normal control (n = 10) samples. Error bars represent mean ± SD. p Values of unpaired two-tailed t test are shown. (D) The correlation between *GGCT* CNV and mRNA in TCGA lung adenocarcinoma (LUAD) samples (n = 511).

The mRNA and protein expression of *GGCT* was already known to be up-regulated in various human cancers (Amano et al., 2012, Gromov et al., 2010, Kageyama et al., 2007, Kageyama et al., 2015). We checked *GGCT* mRNA expression in various human cancers and indeed found that the mRNA of *GGCT* was up-regulated in various cancers compared with each control tissue, and in several cancer types this difference reached statistical significance (Figure 2A). Based on expression difference, GGCT can be a key gene responsible for chromosome 7p amplification in human LUAD. The copy number of *GGCT* is systematically investigated in multiple human cancer samples, including lung, prostate, and colon using the TCGA database. Results confirmed *GGCT* copy number variation (CNV) amplification in multiple human cancers including LUAD (Figure 2B). To further evaluate the CNV status of *GGCT* in cancer, we examined *GGCT* CNV in LUAD samples by qPCR. In these LUAD samples, *GGCT* CNV is significantly up-regulated (Figure 2C).

Besides human cancer samples, *GGCT* CNV status was also systematically analyzed in human lung cancer cell lines. Results indicate that *GGCT* CNV is significantly amplified in human cancer cell lines (Figure S3A). We further evaluated *GGCT* CNV in human cancer cell lines by qPCR, the results confirming the up-regulated *GGCT* CNV in human lung cancer cell lines (Figure S3B). *GGCT* CNV and mRNA expression show significant correlation in both samples of human patients with cancer (Figure 2D) and lung cancer cell lines (Figure S3C). This implies that *GGCT* CNV amplification can directly lead to the up-regulated expression of *GGCT* mRNA. In addition, *GGCT* mRNA expression also significantly correlates with GGCT protein level in lung cancer cell lines (Figure S3D).

### *GGCT* CNV, mRNA, and LUAD Patient Prognosis

It is known that *GGCT* mRNA and protein expression are frequently up-regulated in cancers compared with normal control tissues. However, the consequence of *GGCT* up-regulation in cancer prognosis is still not well studied. Here the prognosis of the patient with lung cancer was evaluated based on *GGCT* CNV status. Interestingly, in early-stage (TNM stage I) LUAD, patients with amplified *GGCT* CNV show significantly decreased overall survival (Figure S4A), whereas in late-stage LUAD *GGCT* CNV amplification does not lead to significantly poor prognosis (Figure S4B). This result suggests that *GGCT* CNV amplification may play a specific function in early-stage LUAD. *GGCT* mRNA expression and LUAD prognosis were also evaluated; *GGCT* mRNA shows similar trends as *GGCT* CNV in early-stage LUAD prognosis; the difference does not reach statistical significance based on p < 0.05 (Figure S4C). In late-stage LUAD, the prognosis of *GGCT* mRNA does not show the same trends as in early-stage LUAD (Figure S4D). This could implicate a specific function of *GGCT* mRNA expression in cancer initiation or early-stage cancer progression. Both *GGCT* mRNA and CNV status do not show significant difference between early- and late-stage LUAD (Figures S4E and S4F). And in both stages of LUAD, GGCT mRNA and CNV have similar correlations (Figures S4G and S4H). Interestingly, *GGCT* CNV amplification or mRNA up-regulation shows similar prognosis as chromosome 7p amplification in both early- and late-stage LUAD. This further implicates the critical function of *GGCT* in cancer-associated 7p amplification.

### *GGCT*^*−/−*^ Mouse Generation and Analysis

**Figure 3:**
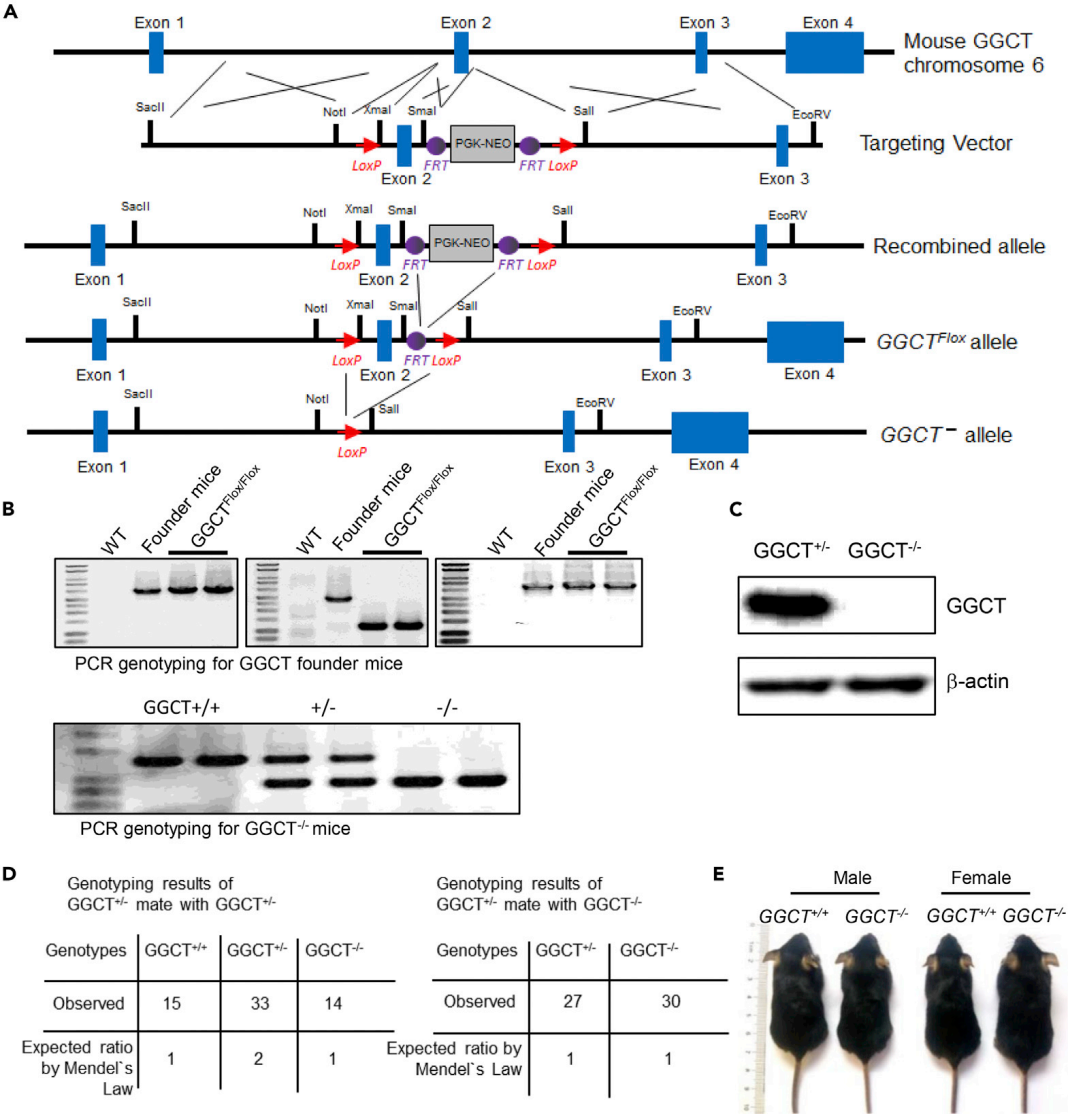
Generation of *GGCT* Knockout Mouse Model and *GGCT*^*−/−*^ Mice Are Viable (A) ES cell-targeting strategy for generating *GGCT* conditional knockout mice. (B) PCR genotyping results for *GGCT* conditional knockout founder mice and *GGCT*^*−/−*^ mice. (C) Western blot analysis of *GGCT*^*+/+*^ and *GGCT*^*−/−*^ MEFs with anti-GGCT antibody; β-actin served as loading control. (D) Genotyping results of *GGCT*^*+/−*^ mouse mated with *GGCT*^*+/−*^ mouse and *GGCT*^*+/−*^ mouse mated with *GGCT*^*−/−*^ mouse. In both cases, the genotype distributions of offspring are in line with Mendel's law, implicating that *GGCT* deletion is compatible with normal mouse embryonic development. (E) Representative pictures of two-months-old male and female *GGCT*^*+/+*^ and *GGCT*^*−/−*^ mice.

No *GGCT* transgenic or knockout mouse model has ever been reported. To further investigate the function of GGCT, we generated *GGCT* conditional knockout (*GGCT*^*Flox/Flox*^) mouse model through embryonic stem (ES) cell targeting and blastocyst injection (Figure 3A). We obtained complete *GGCT* knockout (*GGCT*^*−/−*^) mouse by crossing *GGCT*^*Flox/Flox*^ with EIIα-Cre mouse. The genotyping strategy is described in the Methods section, and genotyping results are shown (Figure 3B). The depletion of GGCT protein in *GGCT*^*−/−*^ MEFs was further confirmed by western blot analysis with anti-GGCT antibody (Figure 3C). *GGCT*^*+/−*^ mice are viable and show no apparent phenotypes. When *GGCT*^*+/−*^ mice are crossed with *GGCT*^*−/−*^ or *GGCT*^*+/−*^ mice, the genotype of the pups show Mendialian distribution (Figure 3D). Adult *GGCT*^*−/−*^ mice do not show difference in body weight when compared with wild-type control mice of similar ages (Figure S5). This indicates that *GGCT* deficiency is compatible with normal mouse development (Figure 3E).

### *GGCT* Depletion Suppresses Primary MEF Transformation and Lung Cancer Formation in *Kras G12D* Mouse Model

**Figure 4:**
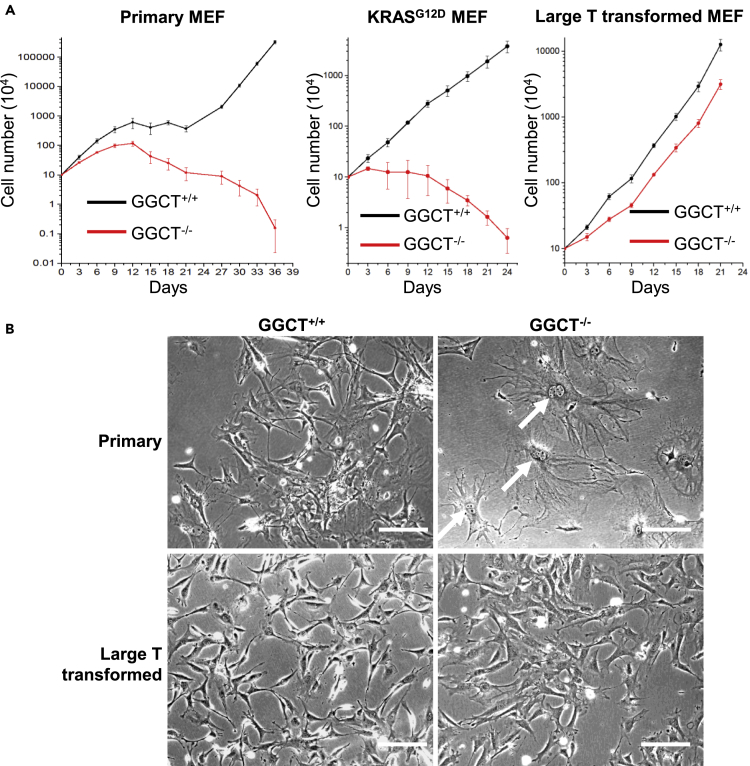
*GGCT*^*−/−*^ MEFs Show Proliferation Inhibition, Senescence, and Resistance to Automatic Transformation Phenotypes (A) Cell proliferation assay. 10 × 10^4^ MEFs of indicated genotypes are seeded into 3.5-cm tissue culture dishes, and the cell numbers are counted every 3 days. Error bars represent mean ± SD of three experiments. (B) Phase contrast images of primary and large T-transformed wild-type and *GGCT*^*−/−*^ MEFs. Nearly all late-passage (P7) *GGCT*^*−/−*^ MEFs are senescent, whereas same-passage primary wild-type or transformed wild-type, GGCT ^−/−^ MEFs do not show significant senescent features. White arrows indicate typical senescent cells, which show flat morphology and double nuclei phenotypes. Scale bar, 50 mm.

To further investigate the function of GGCT in primary cell proliferation and transformation. We isolated and cultured *GGCT*^*−/−*^ and sibling control *GGCT*^*+/+*^ embryonic day 13.5 (E13.5) MEFs from pregnant *GGCT*^*+/−*^ female mouse. Early-stage (before passage 4) *GGCT*^*−/−*^ MEFs can proliferate albeit at slightly slower speed, whereas late-stage *GGCT*^*−/−*^ MEFs show significantly decreased ability in proliferation and completely lose the ability to become automatically transformed during long-passage *in vitro* culture (Figure 4A). *GGCT* deletion also completely blocked the *in vitro* proliferation of *KRAS*^*G12D*^-expressing MEFs (Figure 4A). *GGCT*^*−/−*^ MEFs also show early senescence phenotype (Figure 4B). *GGCT*^*−/−*^ MEFs can be transformed by large T antigen (Figure 4A), which inhibits both Rb and p53 pathways (Ahuja et al., 2005). In large T antigen-expressing situation, both *GGCT*^*−/−*^ and *GGCT*^*+/+*^ MEFs do not show apparent senescent phenotype and can proliferate at similar speed (Figure 4). This indicates that Rb and p53 tumor suppressor may be involved in the growth arrest and senescence phenotypes of *GGCT*^*−/−*^ MEFs.

**Figure 5:**
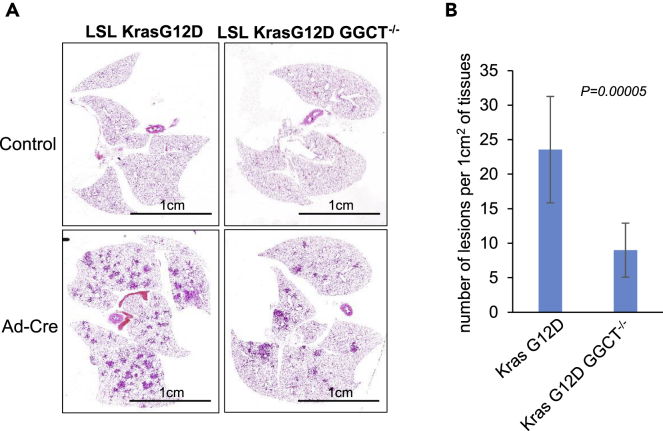
GGCT Is Required for Efficient Cancer Formation in Oncogenic Kras-Driven Mouse Model Lung Cancer (A) Histology of *LSL-Kras G12D* and *GGCT*^*−/−*^*LSL-Kras G12D* mouse lungs 12 weeks after intranasal inhalation of adenovirus Cre. (B) Number of tumor lesions per area of lung tissue in *LSL-Kras G12D* and *GGCT*^*−/−*^*LSL-Kras G12D* mice. Error bars represent mean ± SD of 10 animals. Unpaired two-tailed t test p value is shown. Scale bar, 1 cm.

Our study demonstrated that GGCT transcription is regulated by Ras signaling (Figure 1). To further investigate the *in vivo* function of GGCT in oncogenic Ras-induced cancer formation, we crossed *GGCT*^*−/−*^ mouse with (Lox-Stop-Lox) *LSL- Kras G12D* mouse model and induced lung cancer formation through intranasal inhalation of Cre recombinase-expressing adenovirus (Jackson et al., 2001). Significantly decreased tumors are formed in *GGCT*^*−/−*^ *LSL- Kras G12D* mouse compared with *LSL- Kras G12D* mouse 3 months after adenovirus Cre treatment (Figures 5A and 5B). This observation indicates that GGCT is required for efficient lung cancer formation in oncogenic *Kras*-driving cancer.

### Function of GGCT in Alleviating Oncogenic Stress Signaling

To further investigate the molecular mechanism by which GGCT regulates cell proliferation, senescence, and cancer initiation, RNA sequencing (RNA-seq) analysis was performed with primary, *Kras*^*G12D*^-expressing, or large T antigen-transformed *GGCT*^*−/−*^ and GGCT^+/+^ MEFs. The RNA-seq data generated in this study has been deposited in NCBI SRA database with the accession number SRA: PRJNA554607. In both primary and *Kras*^*G12D*^-expressing situations, cell cycle gene signature, specifically Rb-E2F gene signature, is the top different gene signature when comparing *GGCT*^*−/−*^ and GGCT^+/+^ MEFs (Table S3 and Figure S6), whereas in large T antigen-transformed situation, this Rb-E2F signature does not appear (Table S3), and this observation is in line with the fact that large T antigen is able to block Rb tumor suppressor pathway.

**Figure 6:**
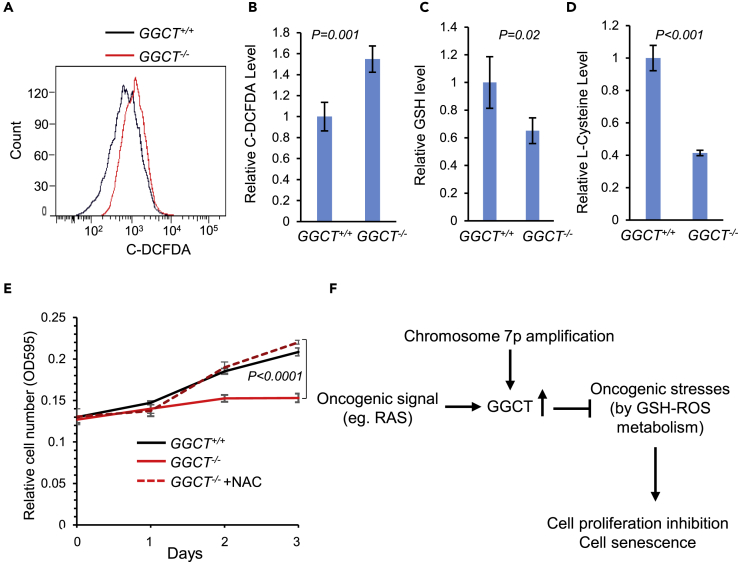
GGCT in Cellular Stress Alleviation (A, B) Cellular ROS level was quantified by carboxy-H2DCFDA flow cytometry in primary wild-type and *GGCT*^*−/−*^ MEFs; error bars represent mean ± SD of four experiments. (C) GSH level was quantified by mass spectrometry in primary wild-type and *GGCT*^*−/−*^ MEFs; error bars represent mean ± SD of three experiments. (D) L-cysteine level was quantified by mass spectrometry in primary wild-type and *GGCT*^*−/−*^ MEFs; error bars represent mean ± SD of three experiments. (E) The proliferation defect of *GGCT*^*−/−*^ MEFs can be rescued by ROS scavenger N-acetylcysteine (NAC, 1 mM) treatment. Error bars represent mean ± SD of three experiments. (F) Proposed model for *GGCT* function in cancer. Both oncogenic signal (like RAS activation) and chromosomal 7p14.3 locus amplification lead to *GGCT* expression up-regulation in human cancers. GGCT helps to alleviate oncogenic stress by regulating GSH-ROS metabolism. In the absence of GGCT, the accumulated cellular stress leads to cell proliferation arrest and cell senescence. For all comparisons, p values of unpaired two-tailed t test are shown.

*GGCT* deficiency has no apparent effect on mouse embryonic development and tissue function (Figure 3). In contrast, during *Kras*^*G12D*^ oncogenic transformation processes, the accumulated cellular stresses need the presence of GGCT to become adapted, and consequently loss of *GGCT* significantly impaired cell proliferation in these situations (Figure 4). Cellular stress represented by reactive oxygen species (ROS) is known to be accumulated during oncogene transformation process (Behrend et al., 2003), and uncontrolled ROS stress can contribute to cell proliferation inhibition and senescence (Pelicano et al., 2004). We did observe that in *GGCT*^*−/−*^ MEFs, ROS level is significantly up-regulated as measured by flow cytometry with ROS indicator carboxy-H2DCFDA (Figure 6A and B).

GGCT is known to have γ-glutamyl cyclotransferase enzyme activity (Oakley et al., 2008). And in plant, this enzyme activity was reported to participate in GSH homeostasis (Kumar et al., 2015, Paulose et al., 2013). However, the function of GGCT in GSH metabolism has not been reported. We then checked the GSH level in MEFs and observed that *GGCT*^*−/−*^ MEFs have significantly decreased GSH level compared with wild-type sibling control MEFs (Figure 6C). This decreased GSH level could contribute to the elevated oxidative stress observed in *GGCT*^*−/−*^ MEFs, because GSH is an important antioxidant in cells. Our study thus suggests that GGCT alleviates oncogenic stress by regulating GSH-ROS metabolism. The cytoplasmic-localized GGCT (Figures S7 and S8) could contribute to intracellular GSH synthesis by regulating the recycling of GSH synthesis substrates (Paulose et al., 2013). In supporting this hypothesis, we observed that in *GGCT*-deficient MEFs, intracellular L-cysteine level is significantly decreased compared with wild-type control MEFs (Figure 6D). Furthermore, the proliferation defect of *GGCT*^*−/−*^ MEFs can be rescued by ROS scavenger N-acetylcysteine (NAC) treatment (Figure 6E), suggesting that GGCT-regulated GSH-ROS pathway is required for primary cell *in vitro* proliferation. In summary, our comprehensive cancer genomic and mouse model studies indicate that both oncogenic signal (Ras activation) and chromosomal 7p amplification lead to *GGCT* expression up-regulation in human cancers. GGCT can help to alleviate cellular stress by regulating GSH-ROS metabolism. In the absence of GGCT, the accumulated cellular stress leads to Rb activation, and consequently cell proliferation arrest and cell senescence (Figure 6F).

## Discussion

Oncogenic transformation in somatic cells is accompanied by various types of cellular stresses (Solimini et al., 2007). Inability to handle these cellular stresses will lead to cellular senescence (Braig and Schmitt, 2006, Kuilman et al., 2010). Oncogenic stress could also be exploited to selectively kill cancer cells but not normal somatic cells through stress sensitization or stress overload (Luo et al., 2009). However, how to alleviate these oncogenic transformation-associated cellular stresses is still a key question for cancer research. Here we report that GGCT functions downstream of common oncogenic signal (like Ras) and is required for the alleviation of ROS stress during oncogenic transformation. GGCT could modulate ROS balance by regulating GSH synthesis.

GSH is synthesized in the cytoplasm, and the availability of L-cysteine is the key determinant of GSH biosynthesis (Lu, 2013). Before the identification of ChaC1 as the first cytosolic pathway for GSH degradation in mammalian cells (Kumar et al., 2012), GSH was thought to be degraded exclusively in the extracellular space by membrane-bound GGT to cysteinylglycine and γ-glutamyl-amino acid dipeptide (Ballatori et al., 2009). One of the best acceptor amino acids for GGT enzymatic reaction is L-cystine (Thompson and Meister, 1976). In the absence of GGT, intracellular GSH level is down-regulated due to decreased availability of intracellular L-cysteine (Bachhawat and Kaur, 2017, Hanigan, 2014). Based on our experimental data, *GGCT* deficiency also leads to decreased intracellular L-cysteine and consequently GSH down-regulation. Thus membrane-bound GGT and cytoplasmic GGCT could be functionally related in GSH homeostasis by recycling L-cysteine. In supporting this interesting hypothesis, GGT expression was also observed to be up-regulated in various human cancers (Hanigan, 2014).

It has already been reported that GGCT protein or mRNA expression is up-regulated in various cancers, including bladder urothelial carcinoma (Kageyama et al., 2007), breast cancer (Gromov et al., 2010), and osteosarcoma (Uejima et al., 2011). GGCT has also been proposed as a biomarker for cancer (Kageyama et al., 2015). However, it was still not known if GGCT up-regulation is a simple consequence of cancer progression, or GGCT is selected to be up-regulated during cancer initiation and progression, and thus can have cancer-driving ability. Bona fide cancer-driving oncogenes are often selected to be amplified through genetic alterations. Here we provide comprehensive and systematic cancer genomics analysis of *GGCT* gene. Our analysis was based on TCGA, and the results have been verified by performing experiments in human cancer samples or cancer cell lines. We found significant *GGCT* genetic amplification in human LUAD and other cancers, importantly *GGCT,* maybe the target gene for chromosome 7p or 7p14.3 amplification in cancer. The amplification of *GGCT* CNV in LUAD compared with normal lung tissue suggests a selection pressure on *GGCT* amplification during LUAD initiation or progression. CNV amplification directly leads to *GGCT* mRNA and protein up-regulation, implicating a potential oncogenic role of GGCT in LUAD.

In early-stage LUAD, *GGCT* CNV up-regulation is associated with significantly decreased patient prognosis, whereas in late stage of LUAD, the prognosis of patients with *GGCT* CNV amplification is not significantly decreased compared with patients without *GGCT* CNV amplification. Similarly, the prognosis of *GGCT* mRNA expression shows similar trends as *GGCT* CNV. In early-stage cancer, GGCT up-regulation makes a difference in patient prognosis, meaning a specific function of GGCT in early-stage cancer, probably in cancer initiation. Our *in vitro* cell culture experiments support the critical function of GGCT in primary cell transformation. And these data are in line with the cancer genomic analysis data, because somatic cell tumorigenic transformation is usually the initial step in cancer progression.

Here we also reported the generation of the first *GGCT* knockout mouse model. *GGCT* deficiency is compatible with normal mouse development and tissue function, but it is required for primary MEFs' *in vitro* growth and transformation. *GGCT* is transcriptionally regulated by oncogenic Ras signal, and in oncogenic Ras-expressing MEFs, *GGCT* loss strongly blocked cell proliferation. More importantly, using a *LSL-Kras G12D* lung cancer mouse model, we demonstrated a critical role of *GGCT* in oncogenic Ras-induced *in vivo* tumorigenesis. These data suggested that GGCT could be targeted to specifically block cancer cell growth, and at the same time to not interrupt normal tissue function.

In summary, here we provide human cancer genomic evidence supporting the oncogenic function of GGCT. We identify GGCT as a downstream target of oncogenic Ras signaling, and that it functions as oncogenic stress alleviator by regulating GSH-ROS metabolism. *GGCT*^*−/−*^ mouse show normal development; however, *GGCT* deficiency inhibits cancer cell proliferation and primary cell transformation and reduces lung cancer formation in *Kras G12D* mouse model. These observations and mechanism study suggest that the selectively amplified *GGCT* in cancer cells can be a therapeutic target for cancer treatment. Furthermore, the newly identified GSH regulator will have implications for understanding GSH homeostasis and oncogenic stress alleviation.

### Limitations of the Study

There are several limitations for this study. First, the detailed mechanism by which GGCT regulates GSH synthesis is not clear. The role of γ-glutamyl cyclotransferase enzyme activity in this function is also not clear. In addition, the molecular link between GSH-ROS metabolism and cell transformation is still unknown, and this requires further study.

## Methods

All methods can be found in the accompanying Transparent Methods supplemental file.

## Data and Code Availability

The accession number for the RNA-seq data reported in this paper is SRA: PRJNA554607.

## Supplemental Information

Document S1. Transparent Methods, Figures S1–S8, and Tables S1–S3
