# Supplementary material for: Ras Downstream Effector GGCT Alleviates Oncogenic Stress
Source: iScience. 2019 Jul 26;19:256–66. doi: 10.1016/j.isci.2019.07.036 (PMC6700472; doi:10.1016/j.isci.2019.07.036)
Supplement: Document S1. Transparent Methods, Figures S1–S8, and Tables S1–S3 [file mmc1.pdf]

ISCI, Volume *19*

Supplemental Information

Ras Downstream Effector GGCT Alleviates Oncogenic Stress

Zaoke He, Shixiang Wang, Yuanyuan Shao, Jing Zhang, Xiaolin Wu, Yuxing Chen, Junhao Hu, Feng Zhang, and Xue-Song Liu

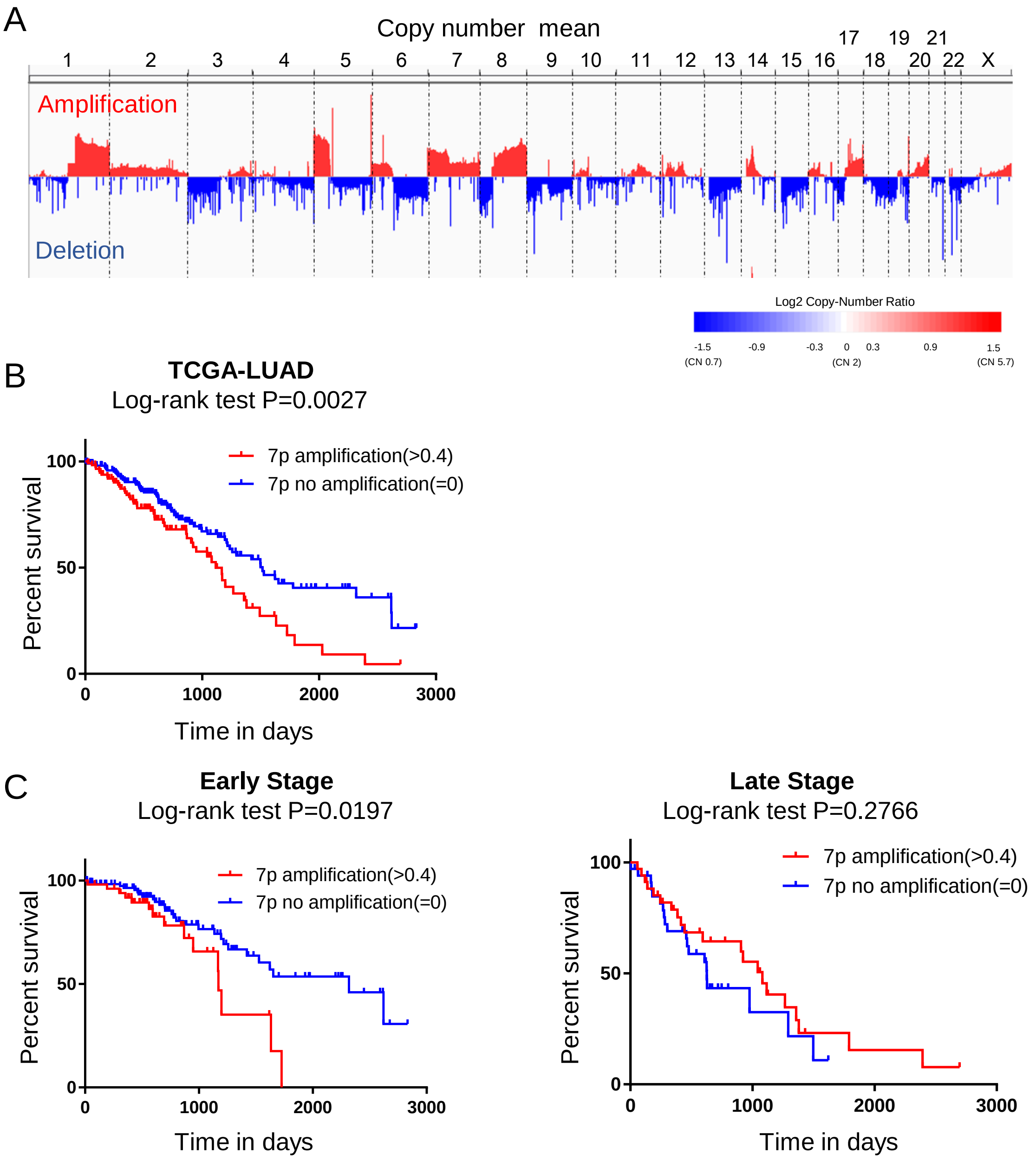

Figure S1. Chromosome 7p amplification and associated patient prognosis in human lung adenocarcinoma (LUAD), Related to Figure 2. (A) Chromosomal level copy number variation status of human LUAD based on TCGA databases.

(B) Kaplan–Meier overall survival curve of LUAD patients with (n=116) or without (n=199) Chromosome 7p amplification are shown. 7p amplification is significantly associated with poor overall survival of LUAD patients.

(C) Kaplan–Meier overall survival curve of early stage (left panel) LUAD patients with (n=51) or without (n=117) chromosome 7p amplification, and also Kaplan–Meier overall survival curves of late stage (right panel) LUAD patients with (n=35) or without (n=34) 7p amplification are shown.

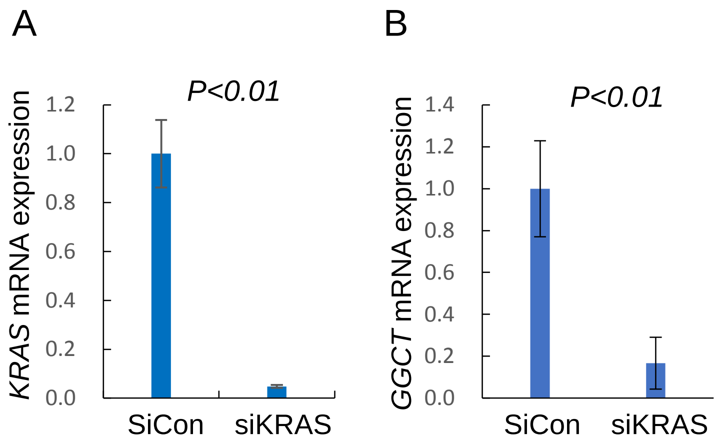

Figure S2. KRAS knockdown leads to decreased GGCT transcription in cancer cells, Related to Figure 1.

KRAS gene was knockdown in A549 lung cancer cells with siRNA. The mRNA expression of *KRAS* (A) and *GGCT* (B) were quantified with qPCR. Error bars represent mean ± s.d. from three experiments.

Chromosome 7p copy number variation

cancer type	Arm	# Genes	Amp frequency	Amp z-score	Amp q-value	Del Frequency	Del z-score	Del q-value
COADREAD	7p	389	0.57	25.2	0	0.02	-6.83	1
COAD	7p	389	0.55	16.3	0	0.02	-7.05	1
GBMLGG	7p	389	0.55	49.1	0	0.02	-5.66	1
GBM	7p	389	0.82	54.1	0	0.07	-1.25	1
KIPAN	7p	389	0.42	24.6	0	0.01	-8.45	1
KIRC	7p	389	0.32	14.7	0	0.01	-6.24	1
KIRP	7p	389	0.6	23.5	0	0.01	-3.91	1
LGG	7p	389	0.23	13.2	0	0.01	-4.83	1
LUAD	7p	389	0.53	9.08	0	0.19	-4.86	1
PRAD	7p	389	0.21	14	0	0	-4.73	1
READ	7p	389	0.62	12.4	0	0.05	-3.35	1
SKCM	7p	389	0.58	12.9	0	0.13	-4.21	1
STAD	7p	389	0.46	10.3	0	0.11	-4.82	1
STES	7p	389	0.5	11.8	0	0.14	-5.68	1
TGCT	7p	389	0.82	11.6	0	0.07	-3.26	1
HNSC	7p	389	0.36	6.01	6.47E-09	0.11	-5.95	1
ESCA	7p	389	0.63	6.06	6.76E-09	0.26	-2.55	1
LUSC	7p	389	0.53	5.72	3.20E-08	0.26	-4.51	1
LIHC	7p	389	0.3	5.06	9.33E-07	0.08	-4.77	1
BLCA	7p	389	0.43	4.9	2.80E-06	0.12	-6.58	1
ACC	7p	389	0.56	4.52	1.06E-05	0.14	-2.63	0.999
DLBC	7p	389	0.33	4.67	3.02E-05	0.06	-0.91	0.984
THCA	7p	389	0.04	4.51	3.26E-05	0	-2.53	0.995
PAAD	7p	389	0.28	4.56	3.47E-05	0.03	-4.07	1
PCPG	7p	389	0.17	4.7	5.38E-05	0.01	-2.9	1
BRCA	7p	389	0.31	3.84	0.000424	0.16	-5.76	1
THYM	7p	389	0.12	3.93	0.000587	0.03	-0.991	0.981
MESO	7p	389	0.29	3.44	0.012	0	-3.36	1
UVM	7p	389	0.11	2.44	0.0275	0	-1.97	0.985
SARC	7p	389	0.35	2.18	0.164	0.2	-2.53	1
KICH	7p	389	0.37	2.19	0.292	0.02	-3.4	1
UCEC	7p	389	0.13	0.265	0.853	0.1	-1.56	1
LAML	7p	389	0.01	-0.907	0.951	0.09	8.96	0
CHOL	7p	389	0.22	-0.0205	0.989	0.14	-1.07	0.982
CESC	7p	389	0.14	-2.82	1	0.1	-4.08	1
OV	7p	389	0.38	-0.625	1	0.32	-3.1	1
UCS	7p	389	0.45	-0.285	1	0.42	-0.635	0.975

Table S1. **Chromosome 7p amplification in various cancer, Related to Figure 2.**

Chromosome 7p copy number variation status in different types of human cancer are shown. For both amplification and deletion, the table has columns for the frequency of amplification (or deletion) of the arm, and associated Z score and Q value.

7p 386 gene

Gene Symbol	logFC >0	AveExpr	t	P.Value	adj.P.Val	B	Locus ID	Cytoband
BZW2	1.409732	6.245525	12.87138	1.16E-26	5.36E-25	49.70866	28969	7p21.1
EIF2AK1	0.972654	7.641871	12.25481	6.16E-25	2.32E-23	45.71858	27102	7p22.1
KDELR2	1.02023	8.261081	11.05695	1.35E-21	3.46E-20	38.06302	11014	7p22.1
CBX3	1.053988	7.407541	10.7314	1.08E-20	2.45E-19	35.98831	11335	7p15.2
DDX56	0.844272	5.975058	9.914304	1.88E-18	3.21E-17	30.88133	54606	7p13
GGCT	1.413713	5.456169	9.896164	2.1E-18	3.57E-17	30.84137	79017	7p14.3
TBRG4	1.046129	5.548764	9.884706	2.26E-18	3.82E-17	30.73586	9238	7p13
ANLN	3.486002	4.231763	9.805604	3.71E-18	6.12E-17	30.54145	54443	7p14.2
PSMG3	1.449531	4.773457	9.350314	6.26E-17	8.8E-16	27.55179	84262	7p22.3
AIMP2	1.113548	4.447288	9.110528	2.73E-16	3.49E-15	26.1025	7965	7p22.1
GARS	0.984559	6.704351	9.034754	4.35E-16	5.41E-15	25.45433	2617	7p14.3
CCT6A	0.977182	7.425921	8.874154	1.16E-15	1.35E-14	24.47984	908	7p11.2
TTYH3	1.483671	6.624684	8.849724	1.34E-15	1.55E-14	24.3525	80727	7p22.3

7p14.3 45 gene

Gene Symbol	logFC >0	AveExpr	t	P.Value	adj.P.Val	B	Locus ID	Cytoband
GGCT	1.413713	5.456169	9.896164	2.1E-18	3.57E-17	30.84137	79017	7p14.3
GARS	0.984559	6.704351	9.034754	4.35E-16	5.41E-15	25.45433	2617	7p14.3
AVL9	1.156238	4.318109	7.928283	3.22E-13	2.64E-12	19.12399	23080	7p14.3
LSM5	0.790194	4.903927	7.057501	4.52E-11	2.79E-10	14.14556	23658	7p14.3
PLEKHA8	0.950119	2.489666	6.933485	8.93E-11	5.32E-10	13.78726	84725	7p14.3
DPY19L1	1.004306	6.383713	6.431107	1.32E-09	6.66E-09	10.71815	23333	7p14.3
NPSR1	2.615909	-4.82135	5.397301	2.33E-07	8.52E-07	6.698606	387129	7p14.3
ZNRF2	0.501314	4.763988	4.926464	2.03E-06	6.43E-06	3.66741	223082	7p14.3
FKBP14	0.43418	3.573499	3.945982	0.000118	0.000281	-0.0602	55033	7p14.3
SCRN1	0.495392	6.361466	3.091289	0.002342	0.004468	-3.09808	9805	7p14.3
CCDC129	1.572301	-2.09954	2.368098	0.019043	0.030643	-3.97082	223075	7p14.3
RP9	0.217461	3.119666	2.03776	0.043178	0.064404	-5.41377	6100	7p14.3
WIPF3	0.575624	-0.92826	1.832223	0.068729	0.098161	-5.24634	644150	7p14.3

Table S2. Chromosome 7p and 7p14.3 gene expression in LUAD, Related to Figure 2.

mRNA expression of chromosome 7p (386 gene) and 7p14.3 (45 gene) genes are ranked based on the statistical difference between normal control and LUAD based on TCGA RNA-seq data.

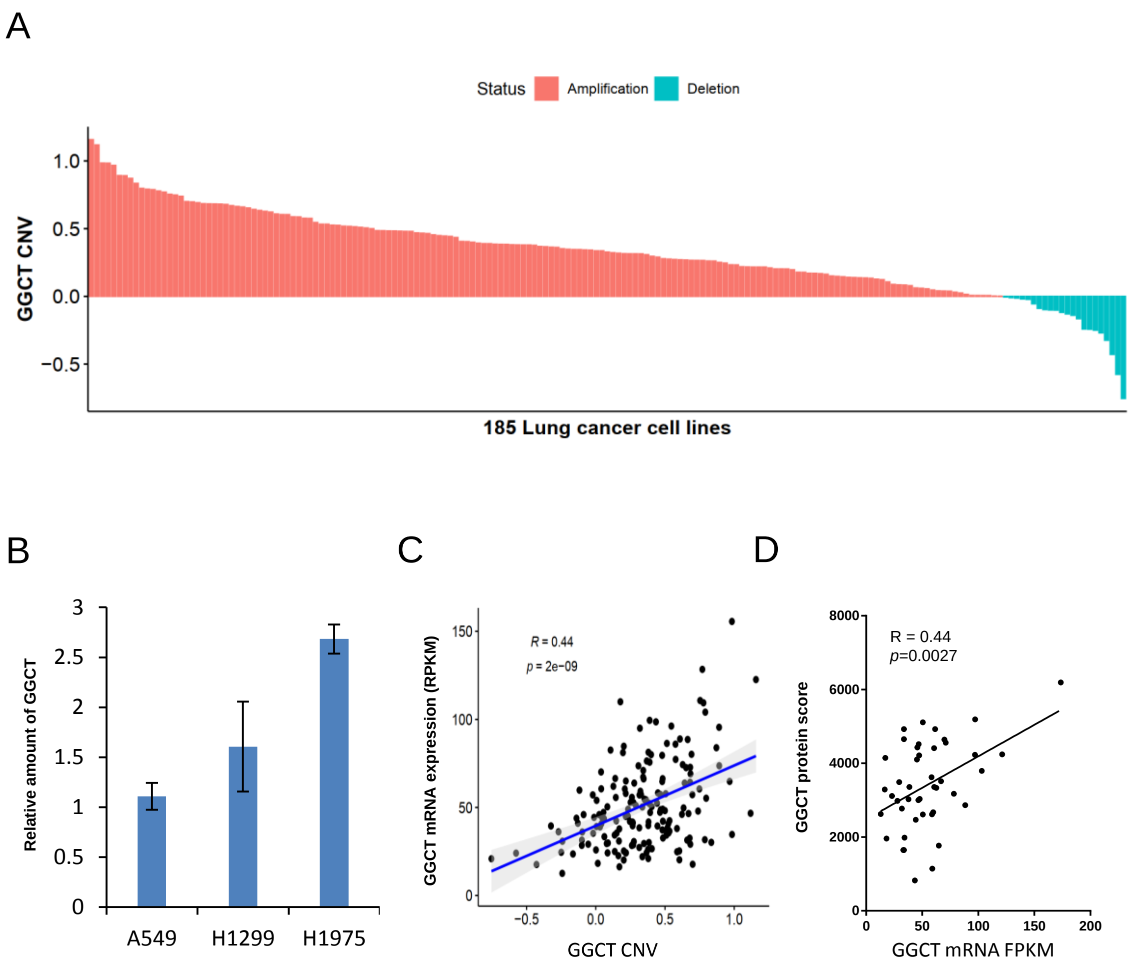

Figure S3. *GGCT* CNV amplification in human lung cancer cell lines, Related to Figure 2.

(A) *GGCT* GISTIC2 CNV values in 185 lung cancer cell lines was shown based on Cancer Cell Line Encyclopedia (CCLE) database. Majority of lung cancer cell lines displayed amplification of *GGCT* locus.

(B) *GGCT* CNV values were quantified by qPCR in three lung cancer cell lines (A549, H1299 and H1975). Error bars represent mean ± s.d. of three experiments.

(C) Correlation between *GGCT* CNV and mRNA expression in lung cancer cell lines (n=172).

(D) Correlation between *GGCT* mRNA and protein expression in lung cancer cell lines (n=44) based on data from the human protein atlas.

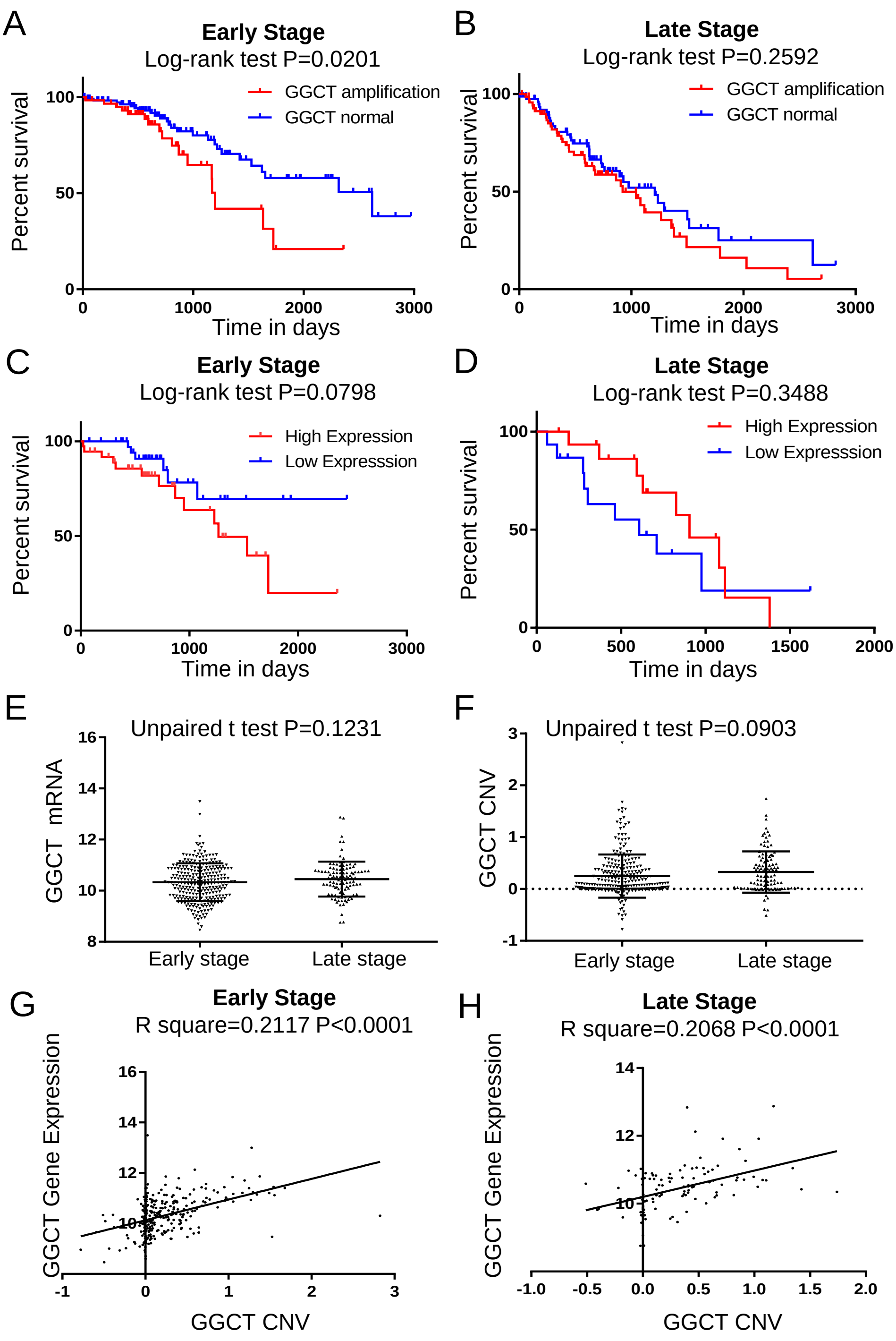

Figure S4. *GGCT* CNV and mRNA up-regulation are associated with poor prognosis in early stage LUAD patients, Related to Figure 2.

(A-D) Kaplan–Meier overall survival curve of LUAD patients are shown. (A and B) Patients are separated into two groups based on *GGCT* CNV value in early stage (A) and late stage (B) LUAD patients. For early stage LUAD, *GGCT* amplification cases contain 60 samples, GGCT no amplification cases contain 114 samples. For late stage LUAD, *GGCT* amplification cases contain 71 samples, *GGCT* no amplification cases contain 78 samples. (C and D) Patients are grouped based on *GGCT* mRNA expression in early stage (C) and late stage (D) lung adenocarcinoma samples. For early stage LUAD, *GGCT* mRNA high expression cases contain 37 samples, *GGCT* mRNA low expression cases contain 40 samples. For late stage LUAD, *GGCT* mRNA high expression cases contain 16 samples, *GGCT* mRNA low expression cases contain 15 samples. Overall survival data was based on TCGA database. Log-rank (Mantel-Cox) test *P* values are shown.
(E and F) *GGCT* CNV (E) and mRNA expression (F) in early stage (n=273) and late stage (n=108) LUAD. (G and H) Correlation between *GGCT* CNV and GGCT mRNA in early stage (n=273) (G) and late stage (n=108) (H) LUAD. Unpaired two-tailed t-test *P* values are shown.

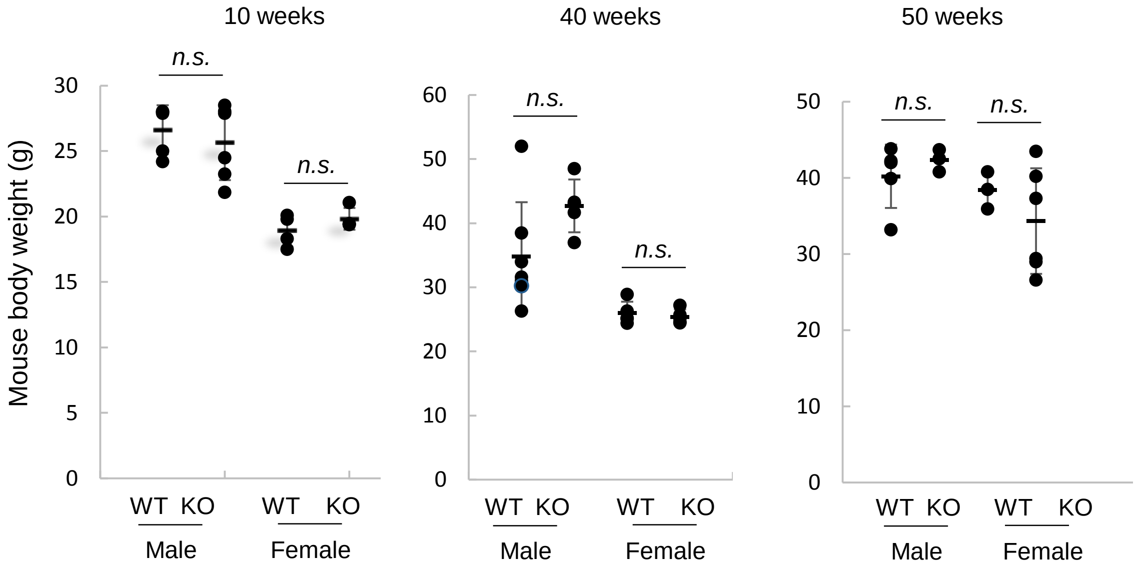

Figure S5. Body weight comparison between *GGCT*$^{+/+}$ and *GGCT*$^{-/-}$ mouse with different ages and sexes, Related to Figure 3.
Male and female *GGCT*$^{+/+}$ (WT) and *GGCT*$^{-/-}$ (KO) mice with different ages are weighted. No significant difference in body weight was observed between *GGCT*$^{+/+}$ and *GGCT*$^{-/-}$ mice.

GGCT$^{-/-}$ vs *GGCT*$^{+/+}$ Primary mouse embryonic fibroblast (MEF)

NAME	NES	NOM p-val	FDR q-val
RB_P107_DN.V1_UP	1.818616	0	0.00479045
E2F1_UP.V1_UP	1.654685	0	0.01618793
GCNP_SHH_UP_LATE.V1_UP	1.564756	0	0.03341101
CSR_LATE_UP.V1_UP	1.476662	0.0039683	0.0574507
HOXA9_DN.V1_DN	1.448844	0.0042553	0.06001841
PRC2_EZH2_UP.V1_UP	1.382798	0	0.09520087
VEGF_A_UP.V1_DN	1.379151	0.0046512	0.08549963
CSR_LATE_UP.V1_DN	1.322314	0.0114504	0.12935296
RPS14_DN.V1_DN	1.310583	0.0080972	0.12997662
ERB2_UP.V1_DN	1.298516	0.0211864	0.13474381

GGCT$^{-/-}$ vs *GGCT*$^{+/+}$ KRAS^{G12D} expressing MEF

NAME	NES	NOM p-val	FDR q-val
RB_P107_DN.V1_UP	1.602945	0	0.172599
CSR_LATE_UP.V1_UP	1.410168	0	0.755149
E2F1_UP.V1_UP	1.336204	0	0.614229
GCNP_SHH_UP_LATE.V1_UP	1.258389	0	1
ERB2_UP.V1_DN	1.216089	0	1
SIRNA_EIF4GI_DN	1.204075	0	1
MEK_UP.V1_DN	1.164132	0	1
VEGF_A_UP.V1_DN	1.160786	0	1
LTE2_UP.V1_DN	1.149189	0	1
HOXA9_DN.V1_DN	1.148979	0	1

GGCT$^{-/-}$ vs *GGCT*$^{+/+}$ Large T antigen expressing MEF

NAME	NES	NOM p-val	FDR q-val
GLI1_UP.V1_UP	1.1033125	0	1
NRL_DN.V1_DN	1.0475771	0	1
SIRNA_EIF4GI_UP	1.0423888	0	1
BCAT_BILD_ET_AL_DN	1.0414152	0	1
CRX_NRL_DN.V1_DN	1.0322751	0	1
CAMP_UP.V1_UP	1.0316353	0	1
ESC_J1_UP_LATE.V1_DN	1.025345	0	1
CSR_LATE_UP.V1_UP	1.0249096	0.169	1
RAF_UP.V1_UP	1.0238483	0	1
PRC1_BMI_UP.V1_DN	1.023609	0	1

Table S3. Differentially expressed gene sets between *GGCT*$^{-/-}$ and *GGCT*$^{+/+}$ MEFs in primary, KRAS^{G12D} expression and large T antigen transformed situations, Related to Figure 4.

Gene set enrichment analysis (GSEA) comparing primary (n=4 for both *GGCT*$^{-/-}$ with *GGCT*$^{+/+}$), *KRAS*^{G12D} expression (n=2 for both *GGCT*$^{-/-}$ with *GGCT*$^{+/+}$), large T antigen transformed (n=2 for both *GGCT*$^{-/-}$ with *GGCT*$^{+/+}$) *GGCT*$^{-/-}$ with *GGCT*$^{+/+}$ MEFs. In each three comparisons, the top 10 enriched gene sets are ranked based on normalized enrichment score (NES) values.

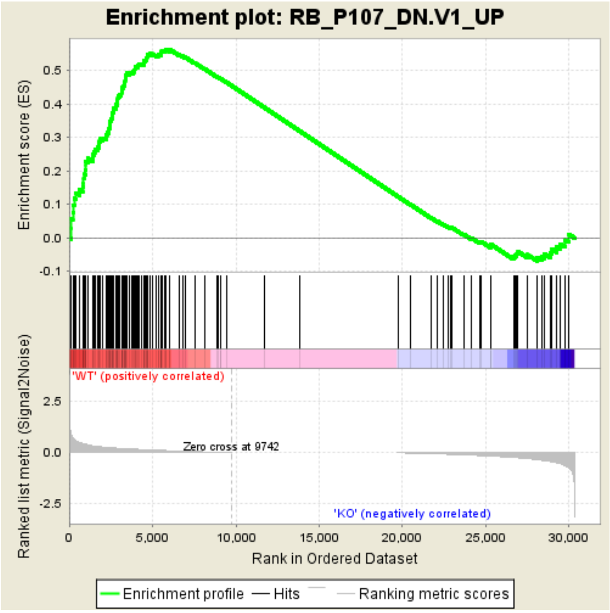

Figure S6. GSEA in primary wild type and *GGCT*$^{-/-}$ MEFs, Related to Figure 4.

RB signature was the top enriched gene signature when compare gene expression difference between wild type and *GGCT*$^{-/-}$ MEFs.

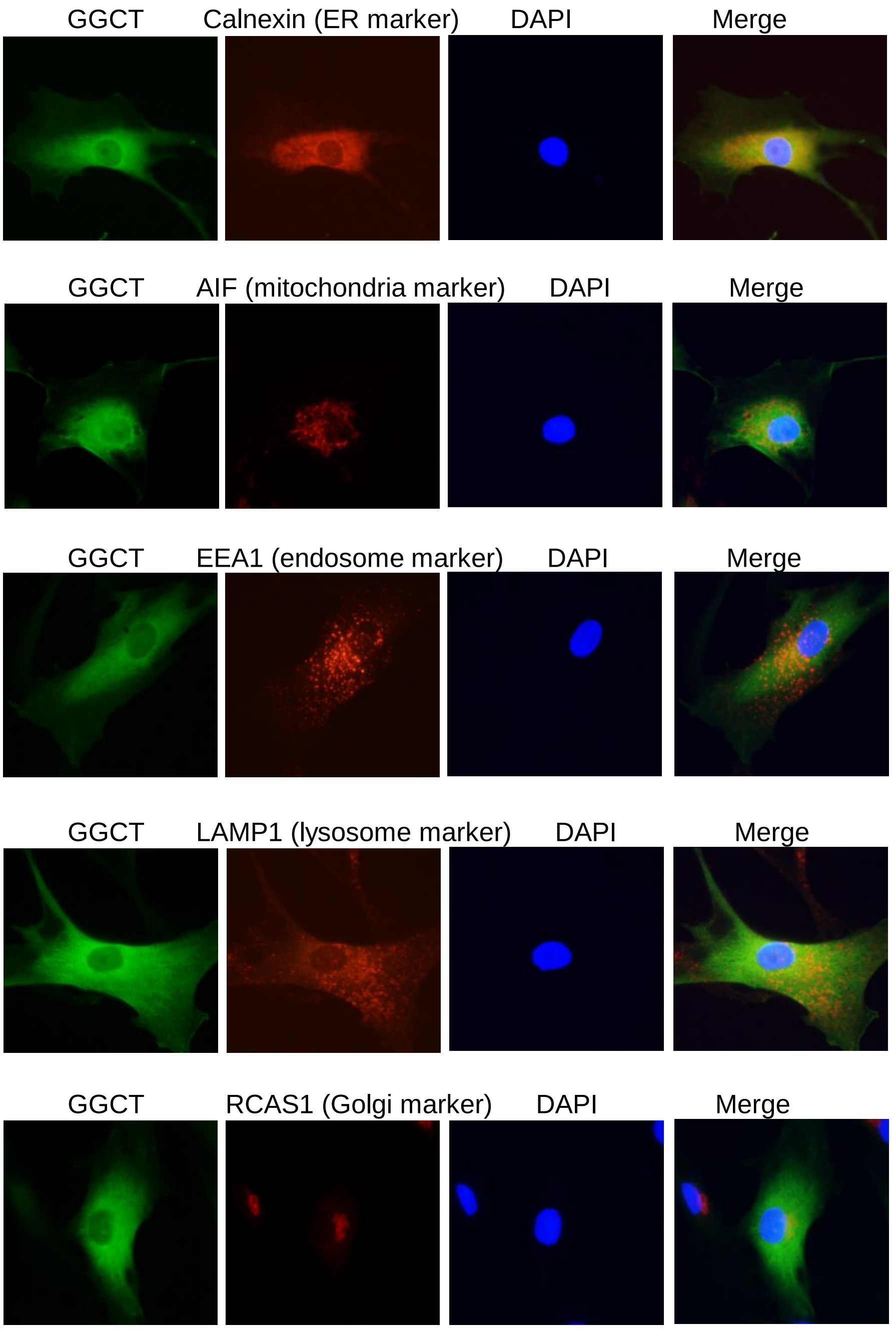

Figure S7. GGCT localization by Immunofluorescence, Related to Figure 6. FLAG tagged GGCT was stably expressed in human fibroblast, localization of FLAG tagged GGCT was determined with anti-FLAG antibody, and co-stained with various cytoplasmic organelle specific antibodies.

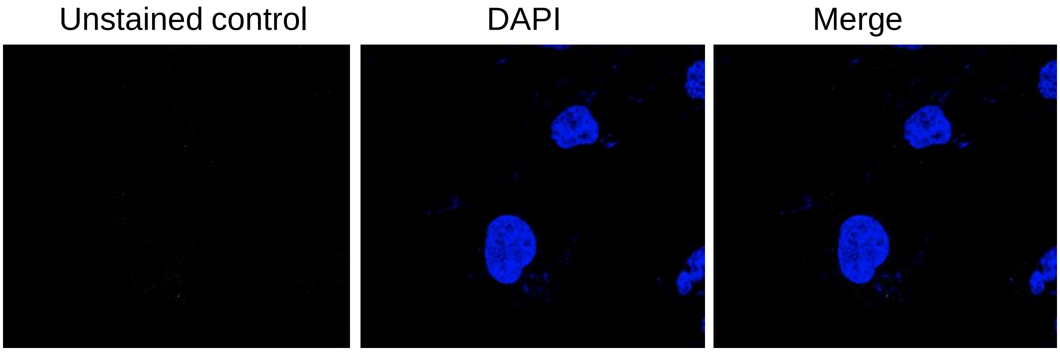

Figure S8. Unstained control for Immunofluorescence, Related to Figure 6. For immunofluorescence staining, the cells was not stained with FLAG antibody, and no background signals can be detected.

TRANSPARENT METHODS

Antibodies and reagents

Anti-GGCT antibody (ab198503, Abcam), Anti-β-actin antibody (AC-15, Sigma), was used for western blot (WB); Anti-FLAG tag antibody (M2, Sigma), Anti- AIF (D39D2, Cell signaling) antibody (C5C9, Cell signaling), Anti- EEA1 antibody (C45B10, Cell signaling), Anti- LAMP1 antibody (D2D11, Cell signaling), Anti- Calnexin antibody (C5C9, Cell signaling), Anti- RCAS1 antibody (D2B6N, Cell signaling) was used for immunofluorescence; Trametinib (Cayman) was used to inhibit MEK signaling, Carboxy-H2DCFDA (ENZO) was used to measure intracellular ROS level.

Human *GGCT* promoter cloning and luciferase reporter assay

Human *GGCT* promoter containing 677-bp upstream of the transcription start site was amplified from human genomic DNA with the primers.

HuGGCT+105 ATGC AAGCTT CCTGAAGCAGAGTGTAAGGAACGG

HuGGCT-677 ATGC CTCGAG TTAAAAAGAGGAAACGGAGACCAGA

The amplified fragment was cloned into the mammalian expression vector pGL3 basic from Promega using the restriction enzymes HindIII and XhoI (New England Biolabs). Luciferase activity was measured 48 h after transfection with the Dual-Luciferase Reporter Assay System (Promega). Values reported are firefly luciferase divided by Renilla luciferase.

shRNA knockdown and qPCR quantification of gene expression

We performed shRNA experiments with lentivirus vector to knockdown *KRAS* genes in human lung cancer cells. KRAS targeting sequence is “5’-ccggcccgttggagctagtggcgtagttcaagagactacgccactagctccaactttttggaaa-3’”.

pLKO.1 vectors with *KRAS* targeting shRNA sequence or control sequence (luciferase targeting) was co-transfected with PSPAX2and pMD2G vectors into 293T cells with Lipo2000 transfection reagent according to the manufacturers’ protocol. After 48 h, the culture medium was filtered through a 0.22 μm filter to

obtain retroviral supernatants. Cells were then infected with the retroviral supernatants and 4 μg/ml polybrene, and after 10 h, supernatants were removed and cells were grown with complete growth medium for an additional 24 h. Infected cells were then selected with 1 μg/ml puromycin. After four days of puromycin selection, cells were used for qPCR quantification analyses. The expression of the *KRAS* and *GGCT* genes was examined by quantitative real-time RT- PCR. Total RNA was extracted from cells using TRIZOL reagent and cDNA was synthesized using 1 μg of total RNA with the HiFiScript gDNA Removal cDNA Synthesis Kit. The primer sequences for *KRAS* mRNA quantification were: 5′ - GGACTGGGGAGGGCTTTCT-3′ and 5′ - GCCTGTTTTGTGTCTACTGTTCT -3′. Primer sequences for *GGCT* mRNA quantification were: 5′ -TGGCAATTCCCAAGGCAAAAC-3′ and 5′ - CCCCTTCTTGCTCATCCAGAG -3′.

Gene copy number detection by qPCR

Lung cancer patients' samples and control blood genomic DNA were provided by Feng Zhang of Quzhou People's Hospital, Quzhou, Zhejiang, China. To validate *GGCT* amplification in cell lines and cancer samples, we carried out qPCR using the UltraSYBR Mixture (Cwbiotech) on an ABI 7500 Real-Time PCR system as per manufacturer's instructions. PCR was initiated at 95°C for 10 min, followed by a 40 cycle amplification (95°C 15 sec, 60°C 1 min). Melting curve analysis was performed to ensure specific PCR product while excluding primer dimers. We used the ΔCT method to calculate relative DNA levels normalized to the mean of internal control gene TUBG1, GAPDH and G6PD. PCR primers are listed below.

Gene	sequence(5'to3')
GGCT CNV-F	AGTGACCAGTACCTTTATTCAGCAT
GGCT CNV-R	GCAATACACAACCAGTTAGTGTGAA
GAPDH CNV-F	AGGGAAGCTCAAGGGAGATAAAATT

GAPDH CNV-R	ATCTAAGAGACAAGAGGCAAGAAGG
TUBG1 CNV –F	AATTTGGAAGCCCAGAGTCTAAGAT
TUBG1 CNV –R	GAAGCAGATAAATCTTGATGGCGAA
G6PD CNV – F	CTATCACTGAATCATAAAACCGTGGG
G6PD CNV – R	TCAAAACCTAAGTGTCTGAGCTATCA

Molecular profile data download and processing

mRNA and protein expression data for Lung cancer cell lines were downloaded from the CCLE portal (http://www.broadinstitute.org/ccle/home). LUAD arm-level copy number variation (CNV) data processed by GISTIC2 (Mermel et al., 2011) were downloaded from the Broad firehose database (http://gdac.broadinstitute.org/). *GGCT* mRNA expression, CNV, and clinicopathological data for TCGA cancer types were downloaded from the UCSC Xena database (http://xena.ucsc.edu/) by R package UCSCXenaTools. In total, GGCT expression level of 32 TCGA cancer types and GGCT CNV status of 31 TCGA cancer types were evaluated. There are 15 types of comparable human cancers (both tumor and normal samples available, and the number of normal samples is greater or equal to 10). GGCT expression levels in 14 of 15 cancer types were statistically up-regulated in tumor samples when compared with normal samples. This data and GGCT CNV status for corresponding cancer types are shown in main Figure 2. Copy number profile was measured experimentally using whole genome microarray at a TCGA genome characterization center. Subsequently, GISTIC2 method was applied using the TCGA FIREHOSE pipeline to produce gene-level copy number estimates. GISTIC2 further thresholded the estimated values to -2,-1,0,1,2, representing homozygous deletion, single copy deletion, diploid normal copy, low-level copy number amplification, or high-level copy number amplification. Genes are mapped onto the human genome coordinates using UCSC xena HUGO probeMap. Of note, gene expression was represented as log2(x+1)

transformed RSEM normalized count unless otherwise specified.

Sample classification strategy

To evaluate the influence of CNV status and gene expression status on LUAD prognosis, GISTIC2 estimated value above 0 (representing diploid normal copy) was defined as amplification, and GISTIC2 estimated value equals to 0 was defined as no amplification. For GGCT gene expression, patients with expression value (log2(x+1) transformed RSEM normalized count) at the top/bottom 20% were classified as High/Low Expression group, respectively.

Cell culture

$GGCT^{-/-}$ and sibling control $GGCT^{+/+}$ mouse embryonic fibroblasts (MEFs) were isolated from embryonic day 13.5 (E13.5) pregnant $GGCT^{+/-}$ female mice mated with $GGCT^{+/-}$ male mice. Lung cancer cell lines A549, H1299, H1975 were obtained from ATCC and confirmed to be mycoplasma free. Mouse embryonic fibroblasts (MEFs), 293T cells were cultured in DMEM (Corning, Cellgro) plus 10% Fetal Bovine Serum (FBS) (Gibco), 100 U/ml penicillin G and 100 µg/ml streptomycin (Corning, Cellgro). A549, H1299, H1975 were cultured in RPMI (Corning, Cellgro) plus 10% FBS, 100 U/ml penicillin G and 100 µg/ml streptomycin. All cells were cultured in 37°C, 5% CO2 incubator.

Generation of $GGCT^{Flox/Flox}$ and $GGCT^{-/-}$ mouse models

$GGCT^{Flox/Flox}$ mouse was generated through ES cell targeting. Targeting vector was based on PGKneoF2L2DTA (addgene plasmid #13445). 5` and 3` arms were amplified from mouse ES cell genomic DNA with the following primers:

5amGgt-USacII, TGCTCTTTTTAGCAGCGCTAGTCC;

5amGgt-DNotI GCTCTAGACTGCTTGCTTTCTCTC;

3amGgt-USalI GTTGTGCGACAGGGTGCCTGATAC;

3amGgt-DEcoRV CTAGATGCAGGATGGCTGGGAGGC;

The replacing exon2 was amplified with following primers:

Exon2Ggt-UXmaI GTGGTATATTGGGATTTAAGGATC;

Exon2Ggt-DSmaI AATTCTACTGTGCTGTTCAATGCC;

The resulting GGCT targeting vector was linearized with SacII and transfected ES cells. The targeted ES clones was initially screened by PCR primers spanning the genomic DNA and inserted cassette, then further confirmed by Sanger sequencing.

Gct5arm-out AGTCATTGCTCTAGACCTTCAGTTT

GGCT5loxp-O GTTCCGGATCCACTAGTTCTAGAGC

Following verification of correct targeting and karyotype, at least two positive ES clones were expanded and injected into blastocysts for mouse generation. The obtained chimeric mouse lines were crossed to C57BL/6J lines for germline transmission. The recombinant founder mice was crossed with ACTB-Flpe mice to get Neo cassette deleted $GGCT^{Flox/+}$ mice. $GGCT^{Flox/+}$ mice crossed with EIIα-Cre mice to get $GGCT^{+/-}$ mouse. $GGCT^{+/-}$ mouse intercrossed to obtained $GGCT^{-/-}$ mouse. $GGCT^{Flox/Flox}$ mice were genotyped with the following primers:

GgctgenoKOFlox-F3a TGAGTCTATGATCTGACAGCAAGAG

GgctgenoFlox-F5a GGAGGGTCACACTTACTAATTGGAT

Predicted PCR product size for wild type allele is 273bp, $GGCT^{Flox}$ allele is 449bp.

$GGCT^{-/-}$ mice were genotyped with the following primers:

GgctgenoKOFLOX-F3a TGAGTCTATGATCTGACAGCAAGAG

GgctgenoKO-F5a ATAACCCCTGTGTAACCATCATTCA

Predicted PCR product size for wild type allele is 994bp, $GGCT^{-}$ allele is 382bp.

$GGCT^{Flox/Flox}$ and $GGCT^{-/-}$ mouse lines were generated by Shanghai Model Organisms Center, Inc. (SMOC). All mouse studies were carried out in strict accordance with the guidelines of the Institutional Animal Care and Use Committee (IACUC) at the School of Life Science and Technology,

ShanghaiTech University.

***LSL-Kras G12D* mouse model and Adenovirus Cre Administration.**

LSL-Kras G12D mouse model was kindly provided by Hongbin Ji of Chinese Academy of Sciences. Both *GGCT*$^{-/-}$ and *LSL-Kras G12D* mice are on C57BL/6 background. 2X10^8 PFU adenovirus containing Cre recombinase or the control virus was instilled into the lungs of 6 to 8 week old *LSL-Kras G12D* or *GGCT*$^{-/-}$ *LSL-Kras G12D* mice as described previously (DuPage et al., 2009). 12 weeks after adenovirus infection, mouse lungs were inflated and fixed with 4% formaldehyde, then paraffin embedded. Lung sections were stained using haematoxylin and eosin (H&E). Images were taken with Olympus VS120 microscope. H&E sections were statistically analyzed by an operator blinded to genotype. Tumor lesion number was quantified using ImageJ software.

GSH, L-Cysteine quantification by Mass-spectrometry

2X10^6 cells were seeded in 10cm dishes. 24 hours later, metabolites were extracted with buffer (80% methanol). Samples were dried in a vacuum concentrator, 200 μL extraction liquid (V acetonitrile: V water= 1:1) was added for reconstitution. LC-MS/MS analyses were performed using an UHPLC system (1290, Agilent Technologies) with a UPLC BEH Amide column (1.7 μm 2.1*100mm, Waters) coupled to Triple TOF 6600 (Q-TOF, AB Sciex). The Triple TOF mass spectrometer was used for its ability to acquire MS/MS spectra on an information-dependent basis (IDA) during an LC/MS experiment. MS raw data (.d) files were converted to the mzXML format using ProteoWizard, and processed by R package XCMS (version 3.2). The preprocessing results generated a data matrix that consisted of the retention time (RT), massto-charge ratio (m/z) values, and peak intensity. R package CAMERA was used for peak annotation after XCMS data processing. In-house MS2 database was applied in metabolites identification.

MEFs virus infection

Primary MEFs were infected with $Kras^{G12D}$ or Large T expressing retrovirus at passage two. Then selected with puromycin (1 μg/ml for $Kras^{G12D}$ retrovirus) or hygromycin (50μg/ml for Large T retrovirus) for three days. The surviving cells after drug selection were harvested for downstream analysis.

Intracellular ROS level quantification by flow cytometry

1.5X 10^5 cells were seeded in each 6 well plate. 24 hour later, Carboxy-H2DCFDA probes (10 μM) was added into culture medium, and cells were stained for 30 min at 37°C. Then staining medium was washed away, the cells were trypsinized, re-suspended and filtered as single-cell solution. Flow cytometry analysis was performed in BD LSR Fortessa Machine. FITC/GFP channel signal was measured. Fluorescent signal data quantification was analysis with FlowJo software.

Immunoblot

Cells were lysed in buffer (50mM Tris, pH8.0, 150mM NaCl and 0.5% NP-40). Protein concentrations of the lysates were measured by Bradford assay. The lysates were then resolved by SDS–PAGE and immunoblotted with the indicated antibodies.

Cell proliferation assay

Cells were seeded in 12-well plates at a density of 10^4/well, then left to grow for four days. Cells were fixed by paraformaldehyde at each time point, and stained with crystal violet. After extensive washing, crystal violet was re-solubilized in 10% acetic acid and quantified at 595 nm as a relative measure of cell number as described previously (Carnero et al., 2000). For serial 3T3 cell proliferation assay, 1.5 X 10^5 cells were seeded into 3.5-cm dishes every 3 days. The cell number was counted with hemocytometer.

RNA-seq analysis

RNA were extracted from primary, *KRAS*^{G12D} or Large T expressing *GGCT*$^{-/-}$ and sibling control *GGCT*$^{+/+}$ MEFs using TRIzol Reagent (Invitrogen, Carlsbad, CA,USA) according to the manufacturer's protocol and then the RNA is quantified by ND1000 Spectrophotometer (NanoDrop Technologies, Wilmington, DE). The cDNA libraries preparation and sequencing were performed by WuXi AppTec according to their standard protocol. Original raw data produced by RNA sequencing were converted to FASTQ files using Illumina CASAVA. We used GSEA (Gene set enrichment analysis) version 3.0 downloaded from Broad Institute to identify the differentially enriched gene signatures between *GGCT*$^{+/+}$ and *GGCT*$^{-/-}$ MEFs. Eight MSigDB gene sets (hallmark gene sets, positional gene sets, curated gene sets, motif gene sets, computational gene sets, GO gene sets, oncogenic signatures, immunologic signatures) were included in our computational analysis. Normalized enrichment scores (NES) were used to rank the differentially enriched gene sets. All RNA-seq data generated in this study has been deposited in NCBI SRA database with the accession number PRJNA554607.

Statistics

The significance of the correlation between *GGCT* CNV, mRNA with clinicopathological characteristics was determined by Student's t-test and fitted with a linear regression model. The significance of the differences between tumor and normal tissues *GGCT* mRNA was tested by unpaired student's t-test assuming unequal sample variance. ANOVA analysis was used when comparing expression levels in more than two groups. Overall survival was estimated using the Kaplan–Meier method with log-rank test. All data were primarily processed by R software (https://www.r-project.org/). Some statistical analyses and visualization were performed using the software GraphPad Prism 7.00 unless otherwise specified. Error bars were presented as means ± SD, and $p < 0.05$ was considered statistically significant.

Supplemental Reference

Carnero, A., Hudson, J.D., Price, C.M., and Beach, D.H. (2000). p16INK4A and p19ARF act in overlapping pathways in cellular immortalization. Nat Cell Biol 2, 148-155.

DuPage, M., Dooley, A.L., and Jacks, T. (2009). Conditional mouse lung cancer models using adenoviral or lentiviral delivery of Cre recombinase. Nat Protoc 4, 1064-1072.

Mermel, C.H., Schumacher, S.E., Hill, B., Meyerson, M.L., Beroukhim ,R., and Getz, G. (2011). GISTIC2.0 facilitates sensitive and confident localization of the targets of focal somatic copy-number alteration in human cancers. Genome Biol. 12, R41.
